# Another challenge for scientists

**DOI:** 10.1186/1476-4598-7-63

**Published:** 2008-07-17

**Authors:** Laura M Christian, Hassan R Naqvi, Christian Schmidt, David Covarrubias, Shawn Mathur

**Affiliations:** 1 Section of Molecular Genetics and Microbiology and Institute for Cellular and Molecular Biology, University of Texas, 1 University Station, A 5000, Austin, TX, 78712, USA; 2 Molecular Cancer, Biomed Central Ltd., Middlesex House, 34-42 Cleveland Street, London, W1T 4LB, UK

## Abstract

By nature, scientists contribute to our understanding of nature and ourselves. As communities undergo significant changes, new challenges are presented. Here, we offer alternative views on recent changes in society.

## 

Affordable and abundant sources of energy allowed society to prosper and use a fraction of the generated surplus to support research and development according to agreed priorities. Available resources allowed the scientific community to bloom and use self-evaluation mechanisms to determine areas of funded research. At this point, involvement of scientists in procedures, designed to allocate a generated surplus to projects, seemed to be of lower priority, given the vast amount of surplus and how little the scientific community needed to prosper. Now, that true costs of energy sources are more clear, much of the generated surplus has to be re-invested to neutralize environmental effects of energy production, distribution et cetera. This reduced overall surplus is mirrored in a decline in research funding with a resulting 'pruning' of projects and declined 'high-risk' areas of research.

As with the advent of an information era and inter-connected civilizations, advances in knowledge are now considered strategic assets, let alone the translation of readily accessible knowledge into (a) more cost-effective neutralization strategies and (b) areas of immediate concern to allow a sustained and affordable generation of energy. The once seen 'luxury spending' on science can now be seen as a central effort of society to stay competitive in the attraction of brightest scholars and creating new areas for lucrative commerce, all of which leading to a windfall of benefits, if not self-enhancing loops.

So, science spending should be brought to the greater attention of the larger community for debate and priority-setting. Failure of scientists to exercise their rights and duties as citizens of a community to raise their voices in the debate and prevent funding from dropping below a critical level may ultimately result in the passing of great chances of a community with the ultimate consequence of declining life-standards in case the speed of progress and participation of knowledge-addition falls below the new and significantly higher average contribution of all nations.

In essence, the new rising information era shifts paradigms and priorities alike, hence, challenging scientists to stay at the forefront of not only path-breaking thinking but also alerting local and global communities about risks and benefits of fostered spending in innovation.

## Conclusion

The burden of providing adequate funds to allow development of new technique and advance knowledge requires combined effort of nations. It remains to be debated whether science for the sake of science or periodical pruning are two extremes or two sides of one coin.

## Competing interests

LMC, HRN, DC and SM declare that there are no competing interests. CS is deputy editor of *Molecular Cancer *and receives no remuneration for his efforts.

## Authors' contributions

CS drafted and finalized this paper. HRN discussed ideas with CS that ultimately resulted in this paper and provided insightful critique. LMC, DC and SM assisted in gathering background information.

